# Application of spectral characteristics of electrocardiogram signals in sleep apnea

**DOI:** 10.3389/fbioe.2025.1636011

**Published:** 2025-07-16

**Authors:** Jiayue Hu, Liu Yang, Xintong Zhao, Haicheng Wei, Jing Zhao, Miaomiao Li

**Affiliations:** ^1^School of Electrical and Information Engineering, North Minzu University, Yinchuan, Ningxia, China; ^2^School of Medical Technology, North Minzu University, Yinchuan, Ningxia, China; ^3^School of Information Engineering, Ningxia University, Yinchuan, Ningxia, China

**Keywords:** sleep apnea, EEMD-ICA, spectrum features, IMF, random Forest

## Abstract

**Background:**

Electrocardiogram (ECG) signals contain cardiopulmonary information that can facilitate sleep apnea detection. Traditional methods rely on extracting numerous ECG features, which is labor-intensive and computationally cumbersome.

**Methods:**

To reduce feature complexity and enhance detection accuracy, we propose a spectral feature-based approach using single-lead ECG signals. First, the ECG signal is preprocessed via ensemble empirical mode decomposition combined with independent component analysis (EEMD-ICA) to identify the most representative intrinsic mode function (IMF) based on the maximum instantaneous frequency in the frequency domain. Next, Hilbert transform-based time-frequency analysis is applied to derive the component’s 2D time-frequency spectrum. Finally, three spectral features—maximum instantaneous frequency (f_emax_), instantaneous frequency amplitude (V), and marginal spectrum energy (S)—are quantitatively compared between normal and sleep apnea populations using an independent-sample t-test. These features are classified via a random forest machine learning model.

**Results:**

The f_emax_ and IMF7 components of the reconstructed signal exhibited statistically significant differences (p < 0.001) between normal and sleep apnea subjects. The random forest classifier achieved optimal performance, with 92.9% accuracy, 86.6% specificity, and 100% sensitivity.

**Conclusion:**

This study demonstrates that spectral features derived from single-lead ECG signals, combined with EEMD-ICA and time-frequency analysis, offer an efficient and accurate method for sleep apnea detection.

## 1 Introduction

Sleep is a vital physiological process, and its disruption has been linked to various disorders, including respiratory diseases and diabetes (Gross et al., 2006). Clinically, polysomnography (PSG) and electroencephalography (EEG) are standard tools for diagnosing sleep apnea. However, PSG is costly, and multi-channel EEG measurements may disrupt natural sleep patterns (Sun et al., 2021), creating a need for a low-cost, non-invasive alternative. ECG signals present a viable alternative, as they capture respiratory and cardiac patterns associated with sleep-related disorders (Faust et al., 2016). Methods for ECG-based sleep apnea detection can be divided into two main categories: deep learning-based approaches and feature-based machine learning techniques.

Deep learning methods use raw ECG waveforms, relying on neural networks to automatically extract discriminative features. For instance, Li et al. (2018) introduced an unsupervised deep neural network combined with support vector machine (SVM) and artificial neural network (ANN) classifiers to detect apnea events. Similarly, Chang et al. (2020) developed a 1D convolutional neural network (CNN) for single-lead ECG analysis, while Zarei et al. (2022) integrated CNN and long short-term memory (LSTM) networks to classify apnea episodes using the apnea-hypopnea index (AHI). Other studies, such as those by Wang et al. (2019) and Ye et al. (2021), extracted features from RR intervals or frequency bands to improve detection accuracy. Wang et al. (2022) improved feature learning by adding a residual attention mechanism. Although effective, these deep learning models require large datasets, are highly sensitive to noise, and lack interpretability in terms of the physiological basis of apnea.

Feature-based machine learning approaches use manually extracted time-domain, frequency-domain, or statistical features. Song et al. (2015) combined EDR and RR-interval features with a hidden Markov model (HMM), achieving 86.2% accuracy. Bozkurt et al. (2020) used PCA-based feature selection to reduce 225 ECG-derived features, attaining 85.12% classification accuracy with decision trees and SVM. Razi et al. (2021) employed PCA and linear discriminant analysis (LDA) to refine feature selection, with random forest (RF) and SVM yielding optimal results. Indrawati et al. (2022) extracted RR-interval spectral features, finding ANN classifiers achieved 84.64% accuracy. Tripathy et al. (2020) introduced bivariate cardiopulmonary (CP) signal analysis using FAEMD-derived features, reporting 73.19% sensitivity and 73.13% specificity with SVM and RF. Afrakhteh et al. (2021) proposed methods based on RR intervals, heart rate variability (HRV), and cardiopulmonary (CP) bivariate features, which extract discriminative information with enhanced noise robustness. However, these approaches mainly rely on high-dimensional statistical features, which require dimensionality reduction and lead to computationally intensive processes.

To address these limitations, we propose a novel approach leveraging spectral features derived from the maximal instantaneous phase and frequency of ECG signals. First, ensemble empirical mode decomposition with independent component analysis (EEMD-ICA) is applied to enhance signal denoising while preserving intrinsic features. Next, the most representative intrinsic mode function (IMF7) is identified based on its instantaneous phase-frequency characteristics, and its marginal spectrum is quantitatively analyzed to extract three key apnea-related features: (1) maximal instantaneous phase and frequency, (2) corresponding amplitude, and (3) characteristic energy. Finally, random forest (RF) classification validates the discriminative power of these features. This method eliminates redundant feature extraction, reduces computational complexity, and improves detection accuracy by focusing on physiologically relevant spectral properties.

## 2 Materials and methods

**FIGURE 1:**
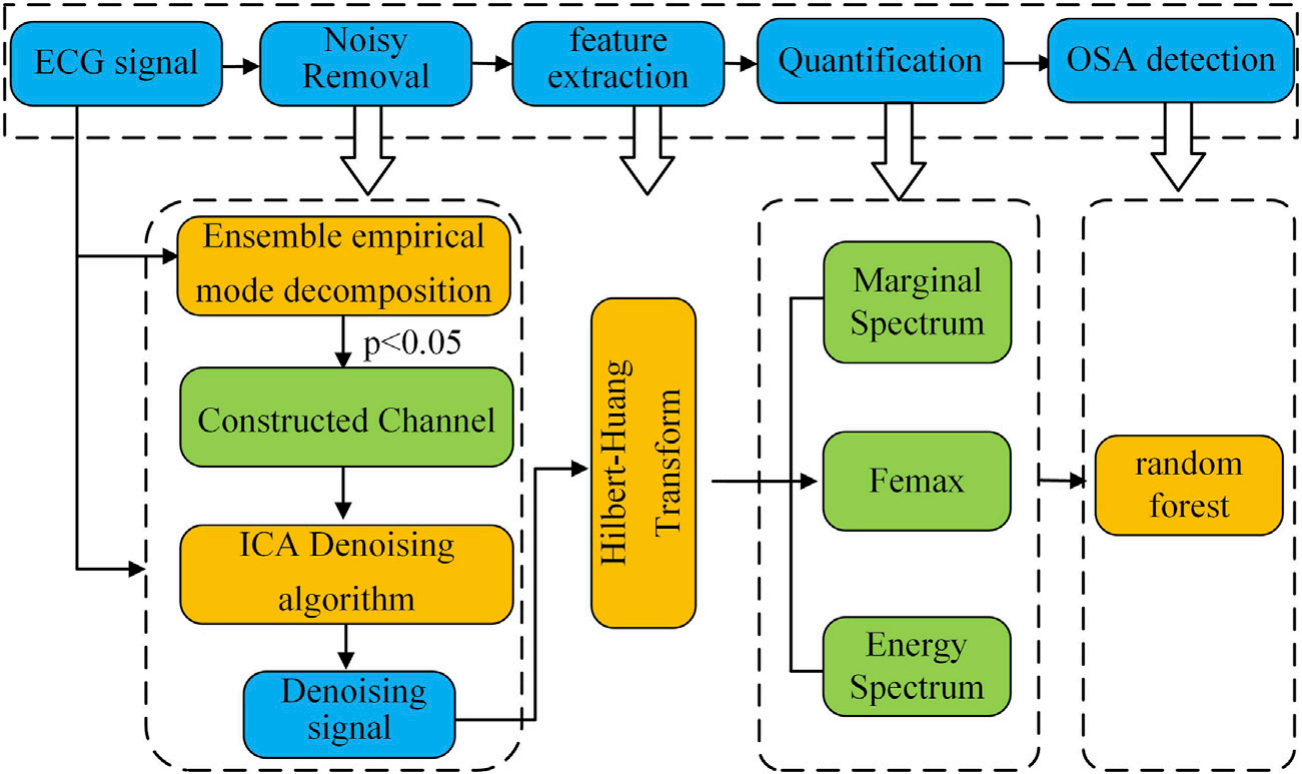
Block diagram of ECG signal spectrum feature classification method.

Figure 1 presents a block diagram of the proposed method for classifying sleep apnea based on the spectral characteristics of ECG signals. The workflow consists of four key stages: (1) ECG signal preprocessing, (2) feature extraction, (3) quantitative feature analysis, and (4) sleep apnea detection.

In the first stage, the raw ECG signal is preprocessed using a combined approach with ensemble empirical mode decomposition (EEMD) and fast independent component analysis (FastICA). This step removes low- and high-frequency noise along with baseline drift, producing a clean ECG signal for further analysis.

Next, the preprocessed ECG signal is decomposed via EEMD to extract intrinsic mode functions (IMFs). Selected IMFs then undergo Hilbert-Huang transform (HHT) to generate the time-frequency spectrum. We then measure instantaneous time-frequency features—including maximum instantaneous frequency and maximum frequency amplitude—along with IMF energy characteristics to create distinguishing spectral parameters. Finally, we test these quantitative features’ effectiveness in separating healthy subjects from sleep apnea patients using three machine learning classifiers.

### 2.1 Data acquisition

This study employed ECG data from the PhysioNet Apnea-ECG Database (Penzel et al., 2000), which contains 70 single-lead recordings classified into three groups based on apnea duration: Group A (≥100 min of apnea events, n = 20), Group B (5–99 min, n = 10), and Group C (<5 min, n = 40). All recordings were approximately 8 h in duration with a 100 Hz sampling frequency. Expert annotators labeled each 1-min segment of the ECG signals, with segments containing ≥1 apnea event marked as “A” and normal breathing segments as “N”.

To minimize borderline cases’ influence, we excluded Group B and focused on 120 recordings (60 from Group A and 60 from Group C), comprising approximately 57,600 1-min segments. The segment distribution showed distinct patterns: Group A contained an A:N ratio of ≈1.8:1 (apnea-dominant), while Group C showed ≈1:19.3 (normal-dominant). The dataset was randomly partitioned at the recording level into training (84 recordings, 40,320 segments) and testing sets (36 recordings, 17,280 segments) in a 7:3 ratio, strictly preserving the original apnea-normal proportion characteristics in both subsets.

### 2.2 Data preprocessing

The acquisition of ECG signals is frequently contaminated by multiple noise sources, which can be categorized as: Low-frequency baseline wander (0–0.5 Hz), Broadband electromyographic interference (5–2000 Hz), Narrowband power line interference (>50 Hz) (Satija et al., 2018).Conventional denoising approaches based on EEMD typically concentrate significant noise energy in the initial IMF components (Dan et al., 2022). While standard practice involves discarding these noisy IMFs, this procedure inevitably eliminates some diagnostically relevant signal components, thereby compromising subsequent feature extraction and analysis.

**FIGURE 2:**
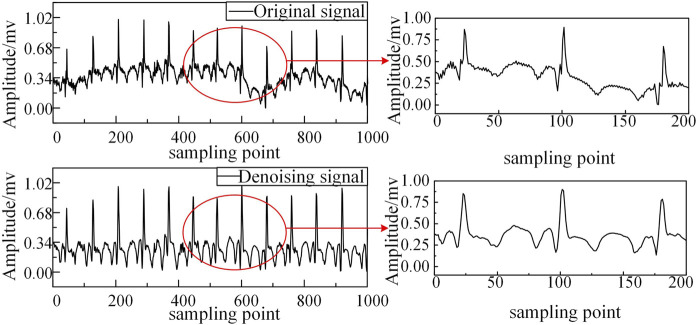
Comparison before and after denoising.

To address this limitation, we employ Independent Component Analysis (ICA), which exploits the statistical independence and non-Gaussian characteristics of source signals to achieve superior noise separation (Ramkumar et al., 2021). Our hybrid EEMD-ICA approach demonstrates three key advantages, as illustrated in Figure 2: Effective suppression of multiple noise sources while preserving signal morphology; Significant reduction of baseline wander artifacts; Enhanced signal quality for subsequent HHT processing.

### 2.3 Hilbert-Huang transform (HHT)

HHT transform is a Time–frequency analysis method with strong adaptability (Zheng et al., 2021). It can obtain the local and global frequency components of the signal based on the non-stationary and nonlinear characteristics of the signal itself, and determine the relationship between the time-frequency energy of the signal. The HHT transform consists of two parts: Empirical Mode Decomposition (EMD) decomposition and Hilbert transform. To solve the problem of modal aliasing during the EMD decomposition process, the Set EEMD is chosen instead of EMD (Dai et al., 2021). Firstly, the EEMD decomposition algorithm is performed as follows:

(1) Add i-fold Gaussian white noise $v_{i}\left(n\right)$ to the original noisy signal $x\left(n\right)$ to form a new signal $x_{i}\left(n\right)$ to be processed (Equation 1):

$$x_{i}=x\left(n\right)+v_{i}\left(n\right)$$ (1)

(2) Using EMD to decompose the new signal $x_{i}\left(n\right)$ into K pieces IMF, denoted as ${IMF}_{i}^{k}\left(n\right)$ , representing the *K*th component and a residual term R obtained by adding the *i*th white noise;
(3) The influence of white noise can be eliminated by averaging the ${IMF}_{i}^{k}\left(n\right)$ components in step (2). When the margin res is a Monotonic function or conforms to the rules, stop and express the original signal as a series of IMF components and the sum of residuals (Equation 2):

$$x=\sum_{i=1}^{n}{IMF}_{i}+res$$ (2)

Then, select the component IMF with the intrinsic characteristics of ECG signal to conduct Hilbert transform (HT) to obtain the corresponding Spectrogram diagram, also known as Hilbert spectrum (Zhang et al., 2016). The specific Hilbert transform process is as follows:

(4) The definition expression of HHT is given by Equation 3:

$$H\left[P_{i}\left(t\right)\right]=\frac{1}{\pi }\int_{-\infty }^{+\infty }\frac{P_{i}\left(t\right)}{t-\tau }d\tau$$ (3)

In the formula: $H\left[P_{i}\left(t\right)\right]$ is the IMF component after Hilbert transform, $P_{i}\left(t\right)$ represents the intrinsic mode component of the electrocardiogram signal.

(5) Construct the analytic signal (Equation 4) from *P_i_* (*t*):

$$Z_{i}\left(t\right)=P_{i}\left(t\right)+jH\left[P_{i}\left(t\right)\right]=a\left(t\right)e^{j\emptyset \left(t\right)}$$ (4)

(6) Differential the phase of the complex function to obtain the Instantaneous phase and frequency $f_{i}\left(t\right)$ of $P_{i}\left(t\right)$ (Equation 5):

$$f_{i}\left(t\right)=\frac{1}{2\pi }\frac{d\emptyset \left(t\right)}{dt}$$ (5)

(7) Display the Instantaneous phase and frequency on the time-frequency plane to obtain the formula spectrum $H\left[\omega ,t\right]$ (Equation 6):

$$H\left(\omega ,t\right)=Re\sum_{i=1}^{n}a_{i}\left(t\right)e^{j\int \omega_{i}dt}$$ (6)

(8) Spectrum $H\left[\omega ,t\right]$ By integrating the time axis, the marginal spectrum can be obtained (Equation 7) (Arrufat‐Pié et al., 2021):

$$h\left(\omega \right)=\int_{0}^{T}H\left(\omega ,t\right)\text{dt}$$ (7)

The marginal spectrum can reflect the energy distribution of each frequency.

**FIGURE 3:**
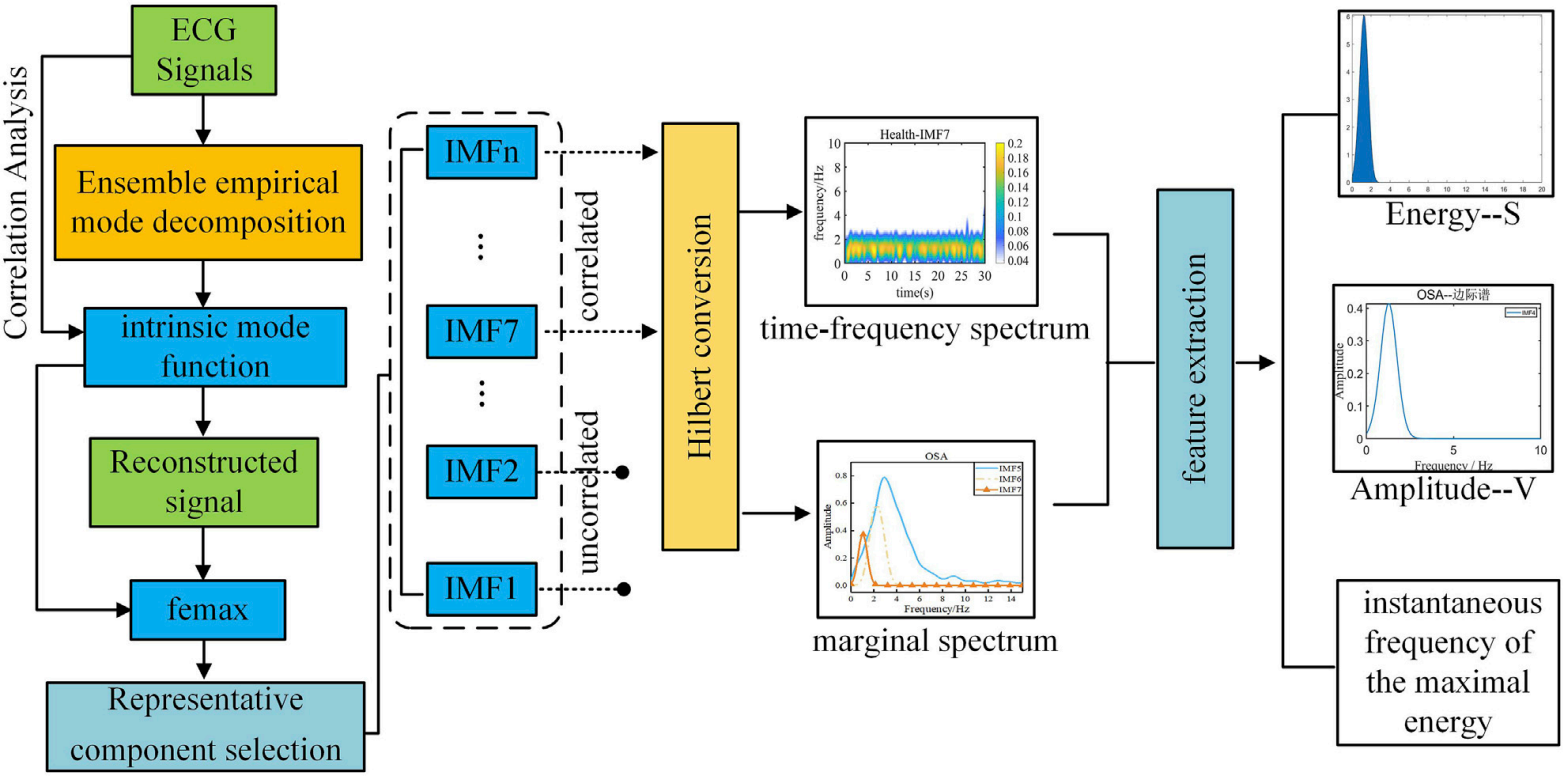
Time frequency conversion diagram of electrocardiogram signal.

The two-dimensional Spectrogram transformation process of ECG signals in this study is shown in Figure 3.

First, the denoised signal is decomposed to extract the intrinsic mode functions (IMFs) of the original signal. Highly correlated IMFs are selected through Pearson correlation analysis and subsequently reconstructed to obtain the refined signal. Next, the maximum instantaneous phase and frequency range (f_emax_) of the original signal is determined by reconstructing its marginal spectrum.

**FIGURE 4:**
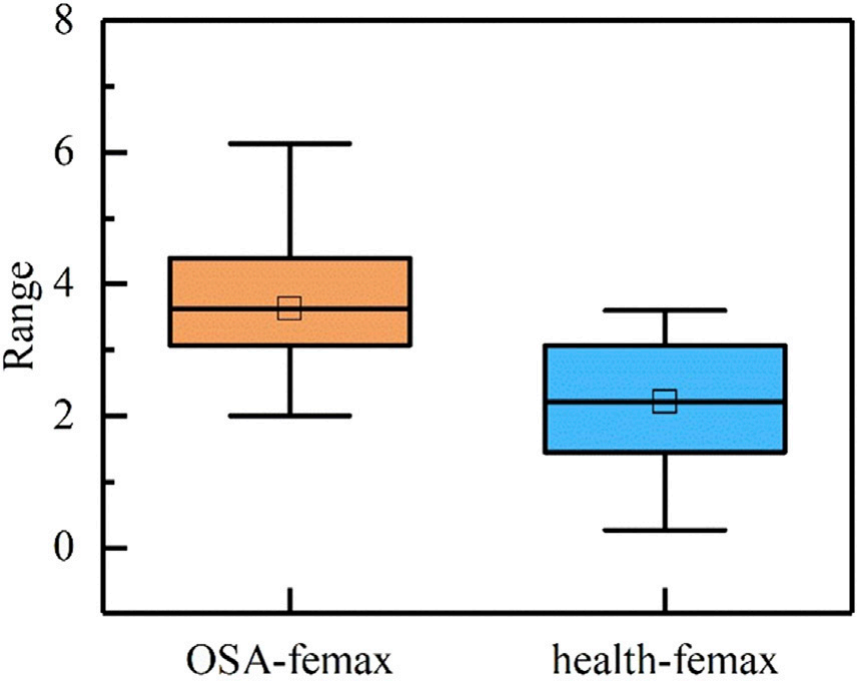
Maximum Instantaneous phase and frequency range of reconstructed signal.

During sleep apnea episodes, significant pathological alterations occur in the frequency and amplitude of cardiac and respiratory activities. By analyzing f_emax_ local variations associated with ECG signals can be characterized. As illustrated in Figure 4, a comparative analysis of f_emax_ between the two groups reveals a distinct divergence in the marginal spectrum’s maximum instantaneous phase and frequency. Specifically, healthy individuals exhibit a concentration near 2 Hz, whereas obstructive sleep apnea (OSA) patients display a predominant frequency around 4 Hz.

Through comparative analysis of the f_emax_ ranges across all intrinsic mode functions and the reconstructed signal, as presented in Table 1, we observed that the most prominent alterations associated with sleep apnea pathophysiology were concentrated in IMF5 through IMF7. We then generated Hilbert-Huang transform time-frequency representations for each component by applying the Hilbert transform to individual intrinsic mode functions.

**TABLE 1:** Femax of each IMF component in healthy individuals and OSA patients.

Health-IMF	Femax	OSA-IMF	Femax
IMF_1_	19.80 ± 4.64	IMF_1_	19.94 ± 4.77
IMF_2_	12.31 ± 1.05	IMF_2_	12.99 ± 1.97
IMF_3_	10.11 ± 0.82	IMF_3_	10.44 ± 1.54
IMF_4_	6.69 ± 0.68	IMF_4_	7.13 ± 0.65
IMF_5_	3.98 ± 1.12	IMF_5_	4.11 ± 1.14
IMF_6_	2.65 ± 0.62	IMF_6_	2.96 ± 0.81
IMF_7_	1.39 ± 0.69	IMF_7_	1.44 ± 0.47
IMF_8_	0.88 ± 0.41	IMF_8_	0.94 ± 0.19
IMF_9_	0.39 ± 0.21	IMF_9_	0.40 ± 0.07
IMF_10_	0.22 ± 0.08	IMF_10_	0.26 ± 0.04

Values are expressed as mean ± SD.

All signal processing and machine learning implementations were performed in MATLAB R2024a (MathWorks, Inc). Specifically, we utilized the Signal Processing Toolbox for EEMD and Hilbert transform computations, and the Machine Learning Toolbox for classification tasks.

## 3 Result analysis and discussion

### 3.1 HHT spectrogram analysis

**FIGURE 5:**
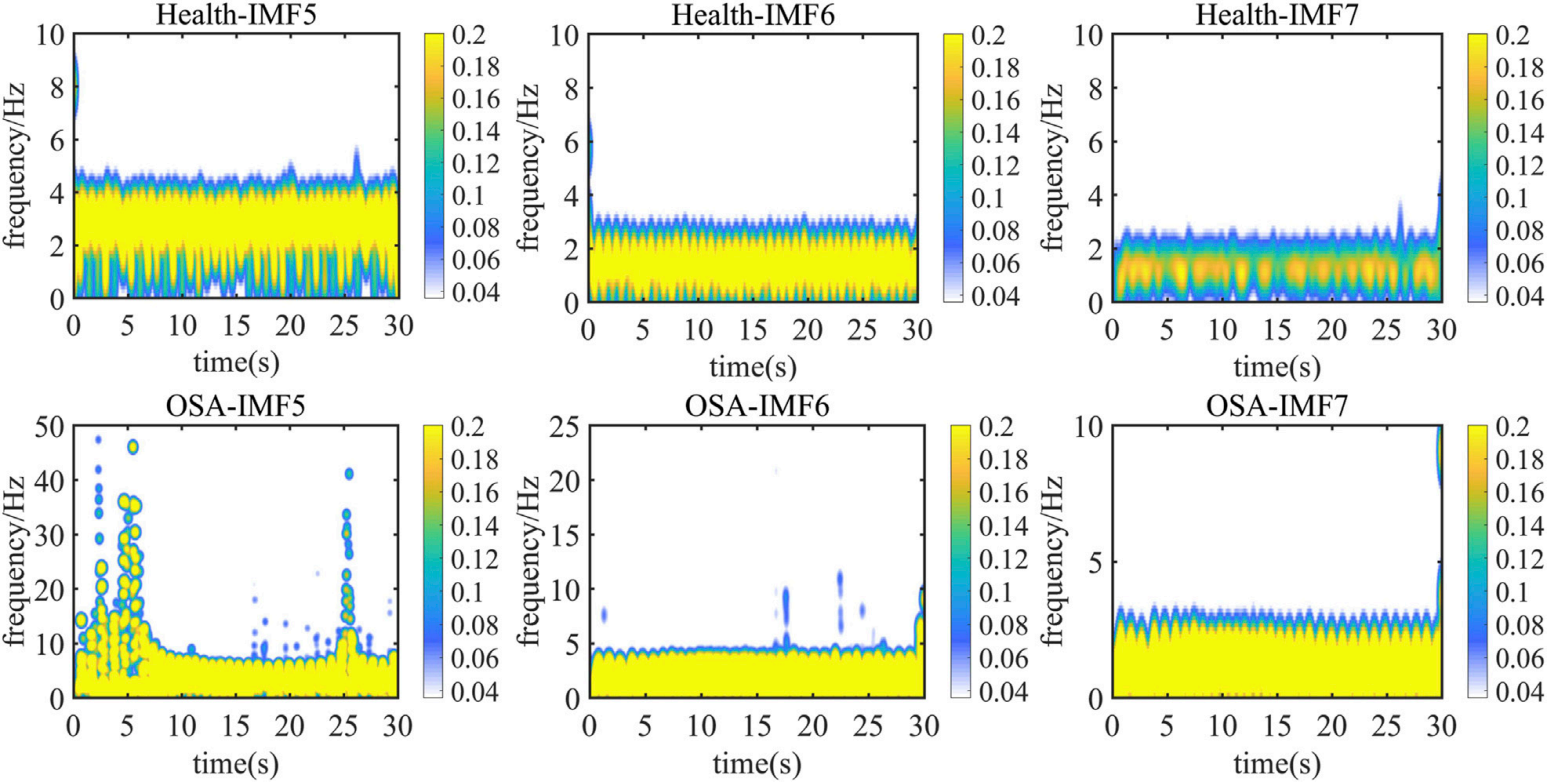
IMF5∼IMF7 Component of Hilbert Spectra.

Figure 5 presents a representative 30-s electrocardiogram signal comparison between normal sleep and sleep apnea conditions. The Hilbert-Huang transform time-frequency distributions of IMF5 through IMF7 components are displayed, with the upper panel showing results from healthy subjects and the lower panel depicting OSA patients. Color intensity corresponds to signal energy concentration at specific time points.

Analysis of energy distribution across frequency bands reveals that while IMF5 and IMF6 components fall within the reconstructed signal’s maximum instantaneous phase and frequency range, their energy distributions exhibit considerable similarity. These components retain some high-frequency noise, making quantitative feature extraction challenging.

In contrast, IMF7 demonstrates the most pronounced intergroup differences. OSA patients show significantly stronger frequency energy concentrations compared to healthy subjects at corresponding time points. This observation suggests that IMF7 captures sleep apnea-related frequency variations, consistent with known physiological effects of hypoxia and sleep disruption on cardiac activity and ECG signal characteristics (Arnaud et al., 2020).

### 3.2 Quantitative analysis of HHT spectrum

**FIGURE 6:**
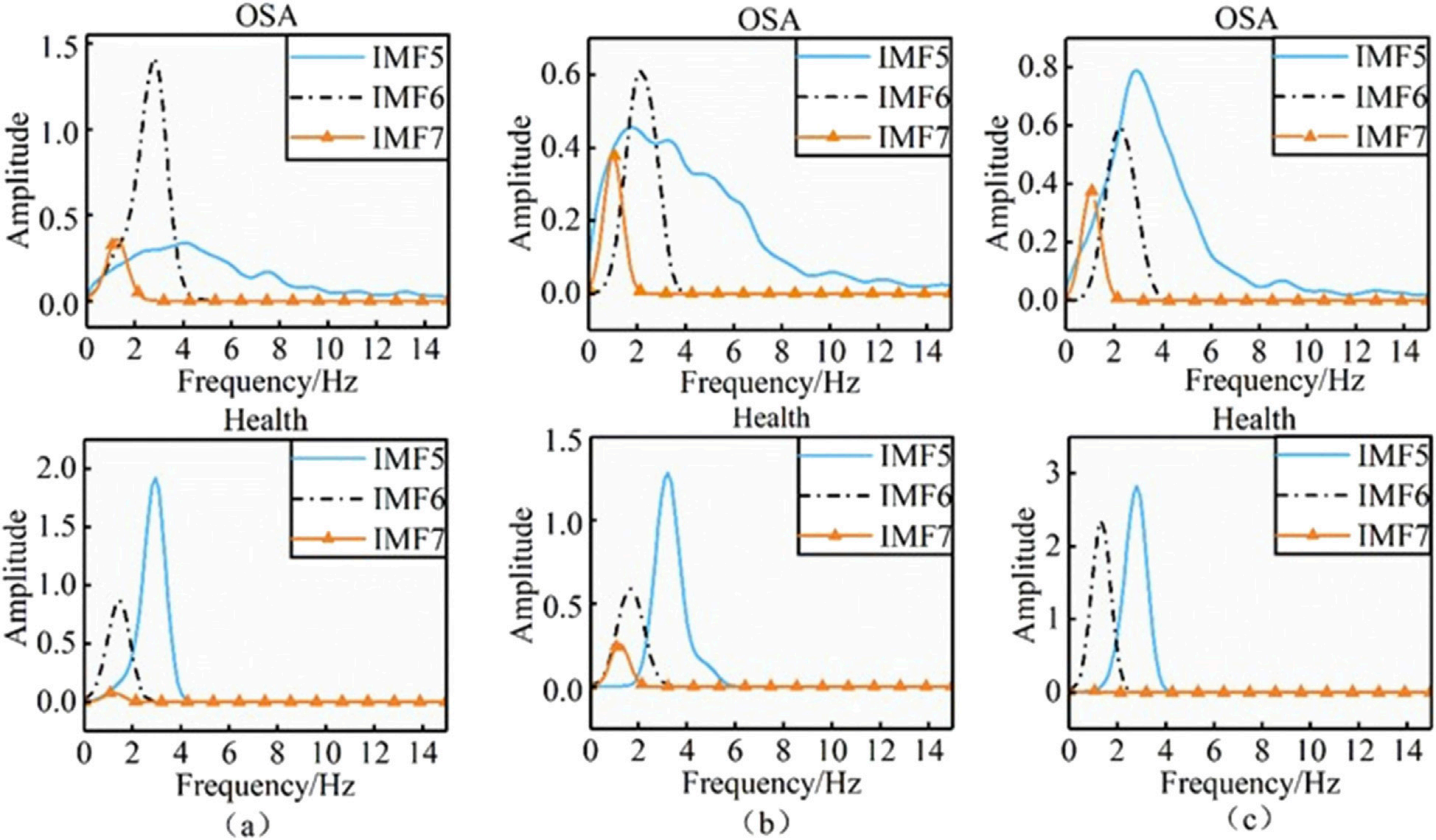
Comparison of marginal spectra between healthy individuals and OSA.

To further quantify the differences in two-dimensional time-frequency maps between healthy individuals and obstructive sleep apnea (OSA) patients, we selected 45 signal sets from healthy subjects in Dataset C and 40 signal sets from apnea patients in Dataset A. The marginal spectra of the intrinsic mode function (IMF) components IMF5 to IMF7 were computed using the Hilbert transform (HT), as illustrated in Figure 6.

Panels (a), (b), and (c) in the figure display the marginal spectra of IMF5, IMF6, and IMF7 for healthy individuals and OSA patients, respectively. Distinct differences in both the shape and amplitude of the marginal spectra were observed between the two groups. To further investigate these spectral differences, we analyzed the following parameters derived from the marginal spectrum in the frequency domain: Maximum instantaneous frequency, Maximum instantaneous amplitude (V), and Characteristic energy (S) (Wei et al., 2018). The characteristic energy S is defined as (Equation 8):

$$S=\int_{\omega_{1}}^{\omega_{2}}h^{2}\left(\omega \right)d\omega$$ (8)

where $w_{1}$ to $w_{2}$ represent the natural frequency range of the marginal spectrum, and $h\left(\omega \right)$ denotes the Hilbert marginal spectrum.

The differences in three groups of indicators between healthy individuals and OSA patients were analyzed using independent sample t-test (SPSS, Windows version 14.0), and the analysis results are shown in Table 2.

**TABLE 2:** Healthy population and characteristic parameters of OSA patients.

Characteristic variable	Health	OSA
femax-Reconstructed signal	2.19 ± 0.99	3.66 ± 1.80**
IMF5- amplitude/V	5.15 ± 2.73	4.56 ± 3.61
IMF5-Characteristic energyS	6.85 ± 3.22	6.21 ± 3.98
IMF5-femax	4.31 ± 1.12	4.11 ± 1.14
IMF6- amplitude/V	5.58 ± 5.96	5.03 ± 5.18
IMF6-Characteristic energyS	6.01 ± 5.83	6.08 ± 5.75
IMF6-femax	2.80 ± 0.62	2.39 ± 0.81
IMF7- amplitude/V	3.17 ± 4.77	2.61 ± 4.41**
IMF7-Characteristic energyS	2.10 ± 3.10	4.89 ± 2.38**
IMF7- femax	1.43 ± 0.69	1.38 ± 0.87

Value expressed as mean ± standard deviation; *p < 0.05:OSA, differs from healthy individuals; **p < 0.001:OSA, has significant differences compared to healthy individuals; Exact p-values for all comparisons are provided in Supplementary Table S1.

The analysis presented in the table reveals that the quantitative parameters of IMF5 and IMF6 show no statistically significant differences between the two study populations. This observation aligns with the patterns observed in the time-frequency maps. In contrast, the IMF7 component exhibits marked differences in maximum instantaneous phase, frequency amplitude, and characteristic energy between groups. Furthermore, the f_emax_ of the reconstructed signal demonstrates significant variation, with healthy individuals consistently displaying lower f_emax_ values compared to patients with obstructive sleep apnea. Patients with OSA conversely show greater amplitude and characteristic energy S values than healthy controls.

This physiological distinction may be explained by the effects of sleep apnea on oxygenation. During apneic episodes, inadequate oxygen supply to the brain and peripheral tissues results in systemic hypoxia, including diminished cardiac oxygenation. In response, the brain initiates compensatory mechanisms such as transient respiratory pulses to elevate breathing frequency, as documented in reference (Hossen and Qasim, 2020). This physiological adaptation consequently increases heart rate, with these pathophysiological changes manifesting clearly in electrocardiogram signals. Through careful examination of the modal and frequency distribution characteristics across ECG signal components, we can effectively differentiate between healthy sleep patterns and those affected by OSA. Of particular note, the IMF7 component combined with the reconstructed signal f_emax_ emerges as a robust discriminative feature for distinguishing normal sleep from sleep apnea.

To assess the diagnostic potential of these features, we implemented a random forest algorithm with stratified 10-fold cross-validation to evaluate classification performance using three distinct feature combinations: IMF5 quantized features with reconstructed signal f_emax_, IMF6 quantized features with reconstructed signal f_emax_, and IMF7 quantized features with reconstructed signal f_emax_.

As demonstrated in Table 3, this study achieved 92.9% classification accuracy with 86.6% specificity and 100% sensitivity using Random Forest, significantly outperforming conventional methods based on heart rate variability or ECG-derived respiration. The developed spectral features demonstrated consistent performance across multiple machine learning architectures, achieving 87.5% accuracy with SVM and 88.24% accuracy with CNN, while preserving physiological interpretability.

**TABLE 3:** Comparison of classification results performance and related work.

Feature	Sen	Spe	Acc
IMF7-femax-RF	**100%**	**86.60%**	**92.90%**
IMF5-femax-RF	85.00%	86.70%	85.90%
IMF6-femax-RF	87.50%	88.90%	88.20%
IMF7-femax-SVM	90.00%	90.00%	87.50%
IMF5-femax-SVM	87.50%	87.50%	87.50%
IMF6-femax-SVM	83.33%	83.33%	86.50%
IMF7-femax-CNN	90.00%	90.00%	88.24%
IMF5-femax-CNN	82.86%	82.86%	82.35%
IMF6-femax-CNN	81.67%	81.67%	82.35%
HRV + EDR (Tripathy and Acharya, 2018)	-	-	78.02%
ECG + EDR (Pinho et al., 2019)	-	-	82.12%

Acc, accuracy; Sen, Sensitivity; Spe, specificit. Bold indicates the column with the best overall results.

To determine the most discriminating component for OSA detection, we performed a systematic feature ablation study using the IMF7 feature set, the results of which are shown in Table 4. The results reveal IMF7-Characteristic energyS as the most critical feature, with its exclusion causing a substantial 28.19% decrease in classification accuracy. The reconstructed signal’s maximum instantaneous frequency also proved highly influential, showing a 22.31% performance reduction when removed. In comparison, amplitude features demonstrated more moderate importance, with a 16.43% accuracy decline upon exclusion. These findings collectively validate our feature selection methodology, as the complete feature ensemble achieved peak performance at 92.90% accuracy by effectively combining these complementary discriminative characteristics.

**TABLE 4:** Feature ablation study on IMF7 components.

Feature combination	Acc	Performance drop
V+S+ femax+ femax-Reconstructed signal	**92.90%**	-
S+ femax+ femax-Reconstructed signal	76.47%	16.43%
V + femax + femax-Reconstructed signal	64.71%	**28.19%**
V + S++ femax-Reconstructed signal	82.35%	10.55%
V+S+ femax	70.59%	22.31%

Notes: Performance drop is calculated relative to the IMF7 quantized features with reconstructed signal femax. Bold values indicate optimal results within each column.

The spectral features extracted from ECG signals in this study offer two significant advantages for OSA detection. First, the time-frequency analysis provides clear physiological interpretation of how OSA affects ECG signals while eliminating irrelevant features. Second, the focused approach on key discriminative components such as IMF7 and femax effectively reduces feature dimensionality. This optimization minimizes the negative impact of excessive features on machine learning performance while maintaining excellent adaptability across different classification algorithms.

## 4 Conclusion

This study identifies four IMF components as characteristic elements of the signal through frequency-domain analysis, specifically using the f_emax_ feature of the original signal. To address the limitations of traditional methods—such as high-dimensional feature spaces and interference from irrelevant features—we analyzed the signal’s frequency energy in the time-frequency domain. As a result, two key features were selected for further analysis: (1) the f_emax_ parameter and (2) the local modal characteristics of the IMF7 component, both of which effectively capture frequency-domain variations associated with sleep apnea.

Building on this, we applied the HHT to derive a two-dimensional time-frequency spectrum of the IMF7 component. This approach provides a more precise representation of the distinctions between normal individuals and sleep apnea patients in the time-frequency domain, clearly showing the impact of sleep apnea on cardiopulmonary activity. However, since visual differences in spectral images are not quantifiable, we further extracted three feature indicators from this component for statistical analysis.

Our findings reveal that the marginal spectral amplitude and the feature energy parameter S of the reconstructed signal (derived from f_emax_ and IMF7) exhibit statistically significant differences between the two groups. To validate the discriminative power of these features, we compared classification performance across multiple algorithms and ultimately selected the random forest model due to its superior accuracy. The results demonstrate that the three proposed features effectively distinguish between sleep apnea patients and healthy individuals, achieving a classification accuracy of 92.9%. Notably, this high detection accuracy was achieved using only three features, outperforming conventional methods.

## Data Availability

Publicly available datasets were analyzed in this study. This data can be found here: https://physionet.org/physiobank/database/apnea-ecg/.
