# Supplementary material for: Application of spectral characteristics of electrocardiogram signals in sleep apnea
Source: Front Bioeng Biotechnol. 2025 Jul 16;13:1636011. doi: 10.3389/fbioe.2025.1636011 (PMC12307459; doi:10.3389/fbioe.2025.1636011)
Supplement: Supplementary file 1 [file Table1.docx]

Table S1 Exact p-values for healthy controls vs. OSA patients' characteristic parameters

| **characteristic variable** | **P-value** |
| --- | --- |
| femax-Reconstructed signal | 3.21×10⁻^4^ |
| IMF5- amplitude /V | 0.187 |
| IMF5-Characteristic energyS | 0.342 |
| IMF5-femax | 0.423 |
| IMF6- amplitude /V | 0.256 |
| IMF6-Characteristic energyS | 0.891 |
| IMF6-femax | 0.052 |
| IMF7- amplitude /V | 8.76×10^-4^ |
| IMF7-Characteristic energyS | 1.05×10^-4^ |
| IMF7-femax | 0.678 |
